# Beverage Consumption Patterns among Overweight and Obese African American Women

**DOI:** 10.3390/nu9121344

**Published:** 2017-12-11

**Authors:** Terryl J. Hartman, Regine Haardörfer, Brenda M. Greene, Shruti Parulekar, Michelle C. Kegler

**Affiliations:** 1 Department of Epidemiology and Emory Prevention Research Center, Rollins School of Public Health and Winship Cancer Institute, Emory University, Atlanta, GA 30322, USA; Shrutiparulekar28@yahoo.com; 2 Department of Behavioral Sciences and Health Education and Emory Prevention Research Center, Rollins School of Public Health, Emory University, Atlanta, GA 30322, USA; regine.haardoerfer@emory.edu (R.H.); mkegler@emory.edu (M.C.K.); 3 Southwest Health District, 8-2, Division of Public Health, Georgia Department of community Health, Albany, GA 31710, USA; bmgreene@dhr.state.ga.us

**Keywords:** beverages, sugar sweetened beverages, overweight, obesity

## Abstract

The goal of this research was to assess patterns of beverage consumption and the contribution of total beverages and classes of beverages to overall energy intake and weight status. We conducted an analysis in a community-based study of 280 low-income overweight and obese African American women residing in the rural South. Participants provided baseline data including demographic characteristics, weight and two 24-h food and beverage dietary recalls. Mean energy intake from beverages was approximately 273 ± 192 kcal/day or 18.3% of total energy intake. The most commonly reported beverage was plain water, consumed by 88.2% of participants, followed closely by sweetened beverages (soft drinks, fruit drinks, sweetened teas, sweetened coffees and sweetened/flavored waters) consumed by 78.9% of participants. In multiple regression analyses total energy and percent energy from beverages and specific categories of beverages were not significantly associated with current body mass index (BMI). It is widely accepted that negative energy balance may lead to future weight loss. Thus, reducing consumption of beverages that contribute energy but not important nutrients (e.g., sugar sweetened beverages) could be an effective strategy for promoting future weight loss in this population.

## 1. Introduction

It is estimated that over one-third of all U.S. adults are obese. However, substantial disparities exist among racial/ethnic minorities [1]. For example, approximately 56.9% of adult African American women are obese compared to 45.7% of Hispanic and 35.5% of Caucasian women [2]. Obesity and weight gain and their accompanying metabolic abnormalities have been linked to several chronic diseases including cardiovascular disease, type 2 diabetes and cancer at several sites [3]. Energy-containing beverages could promote weight gain because they are substantial sources of energy in the U.S. diet [4]. In nationally representative data, Storey and colleagues [5] and more recently, Han and Powell [6] reported that beverage consumption by adults varied by race/ethnicity, socioeconomic status, age and sex. Overall, for women, beverage energy intake peaked at ages 20–39 years contributing approximately 19% of total energy intake, or 380 kcal/day. Han and Powell [6] observed that mean beverage energy intake ranged from 362 kcal/day among Mexican American women and was highest among African American women at 405 kcal/day. Racial minorities were more likely to report consuming more sugar sweetened beverages overall, and among children, adolescents and adults [6]. Added sugar is one factor which could contribute to obesity and related chronic diseases, thus, sugar-sweetened beverages, the largest source of added sugar in the diet of the US population, have been a major focus of obesity prevention efforts [7].

The objective of the present study was to describe the contribution of total beverages and classes of beverages, particularly sugar sweetened beverages, to overall energy intake and to evaluate the associations between beverage consumption and body mass index (BMI) in a population of overweight and obese African American women. 

## 2. Materials and Methods

### 2.1. Population and Data Collection

The Healthy Homes/Healthy Families (HHHF) study is a randomized controlled intervention trial designed to test the effectiveness of home-based coaching to promote healthier home food and physical activity environments for weight gain prevention. Details of the study and data collection procedures have been reported [8]. The trial, developed by the Emory Prevention Research Center (EPRC) in partnership with the Cancer Coalition of South Georgia and the EPRC Community Advisory Board (CAB) followed a community based participatory research model. The CAB is comprised of residents of southwest Georgia representing local organizations such as churches, businesses, health departments, and civic organizations.

All data collection procedures for HHHF were approved by the Institutional Review Board at Emory University and participant verbal informed consent was obtained by telephone. Overweight and obese (body mass index ≥ 25) female participants ages 35–65 were recruited from February 2011 to December 2012 from nine southern Georgia clinic sites affiliated with three federally qualified health centers. Eligible individuals had to speak English, live with at least one other person, and reside within 30 miles of a participating community health center. We excluded those with contraindications for a medically unsupervised intervention that focused on healthy eating and physical activity to promote weight gain prevention (e.g., pregnant women). Trained interview staff collected data by telephone at baseline, six and 12 months. Participants provided demographic (e.g., age, educational status, income, residence, number of children living in the household–defined as sleeping in that home at least three nights per week) and health-related information including physical activity, anthropometry (height in feet and inches and weight in pounds used to calculate body mass index–BMI kg/m^2^), completed a series of home food environment questions, and completed two 24-h dietary recalls (one week and one weekend day) at each time point. The data included in the present report are from baseline, prior to any intervention, and include only overweight and obese African-American participants (*n* = 280).

### 2.2. Dietary Assessment

Dietary data were collected on one weekday and one weekend day on unannounced, non-consecutive days using the Nutrition Data System for Research (NDSR, Version 2010, Nutrition Coordinating Center, University of Minnesota, Minneapolis, MN, USA). Participants were mailed a printed food booklet to assist them when estimating portion sizes for foods and beverages. Data were cleaned by examining 100% of foods, records, and meal quality assurance reports produced by NDSR. Reports were reviewed for irregular food and beverage weights, total energy and fat amounts; database minimum/ maximum amount flags; and food detail notes. Missing foods were resolved by comparing nutrients to product nutrition labels per product websites. Nutrient values were compared to similar NDSR entries and matched by the following set of nutrient tolerances per 100 g of food: 85 kcal; 2.5 g of fat; 100 mg of sodium; 10 g of carbohydrates; 5 g of protein. Results from the two days of intake were averaged. Aggregate beverage groups and nutrients of interest contributed by beverage groups were calculated from the NDSR dietary intake data Food Group Serving Count File and Food File with online guidance from the developers. We grouped beverages into the following categories: 100% fruit/vegetable, milk unsweetened, non-dairy milk, sweetened/flavored milk, sugar sweetened (soft drinks, fruit drinks, sweetened teas, coffees and waters), artificially sweetened, unsweetened (tea, coffee), alcohol, and water (without additions). Non-alcoholic substitutes were rarely consumed and were not included. Meal replacement drinks were considered “food” and were not included. For the aforementioned categories we determined any/no consumption (number, % any) and calculated both volume (mL) and energy (kcal) estimates of absolute intake, percent total beverage volume (%), and percent total energy contribution (%). For analyses, we excluded participants with only one recall or who reported dietary intakes <400 or >5000 kcal total energy/day (adapted from guidelines by Willett [9]).

### 2.3. Statistical Analyses

Statistical analyses were conducted using SAS 9.4 (SAS Institute Inc., Cary, NC, USA). Analyses included 280 participants with complete data. Initial analyses examined the distribution of the beverage and other variables and evaluated distributions and potential outliers. In univariate analyses we evaluated the association between demographic characteristics with total beverage intake as servings, absolute volume (mL), absolute energy (kcal) and percent energy contributed by beverages (%). The associations between demographic and lifestyle characteristics with beverage groups (Table 1) are reported as a percentage of participants who reported consuming any of the category. In subsequent univariate analyses we evaluated the association between groups of beverages with overweight/obesity reflected by categories of body mass index (BMI-kg/m^2^) (Table 2). For these analyses, within each beverage group, we included only those respondents who were consumers. Results are reported as means with standard deviations (SD) (for continuous data) or number and percent (for categorical data) by strata of participant characteristics of interest. Multivariate linear and multinomial regression models were constructed with BMI as the outcome variable coded as continuous and categorically (overweight, obese, morbidly obese), respectively. The results of the multivariate linear and multinomial regression analyses were similar; thus, only the multivariate linear results are presented in results. We evaluated the associations for BMI with total beverage energy variables (continuous total beverage energy (kcal) and % energy from beverages). We also evaluated the associations among BMI with a priori selected grouped beverage variables (continuous 100% fruit/vegetable juices, dairy/non-dairy milks, sugar sweetened beverages, and alcohol) entered into models either as absolute beverage energy (kcal) or percent total energy (%kcal). Results (beta, standard errors (SE) and *p*-values) are reported for all variables under consideration with model fit statistics (total *r*-squared) reported separately. In sensitivity analyses we repeated the above regression analyses for a reduced sample (*n* = 244) which eliminated participants reporting <24 oz. total beverage/day and were considered potential beverage under-reporters. The results for the reduced sample were similar to the overall results; thus, the full sample results are presented throughout. All statistical tests were two-sided and *p*-values < 0.05 were considered statistically significant.

## 3. Results

Table 1 presents total and grouped beverage consumption by demographic and lifestyle characteristics of the population. Overall, in this population of overweight and obese African American women mean energy intake from beverages was approximately 273 ± 192 kcal/day or 18.3% of total energy intake. The most commonly reported beverage was plain water, consumed by 88.2% of participants, followed closely by sweetened beverages (soft drinks, fruit drinks, sweetened teas, sweetened coffees and sweetened/flavored waters) consumed by 78.9% of participants. Unsweetened beverages (tea, coffee, not including water) and artificially sweetened beverages (diet soft drinks, diet fruit drinks, diet tea and diet coffee beverages) were reported by 46.8% and 29.3% of participants, respectively. Milk consumption (without additions) was reported by 68.2% of participants, overall. Study participants who were younger, lived with children, and had more education tended to report greater energy intakes from beverages either for absolute intake (kcal/day) or relative to total energy intake (i.e., percentage of energy intake). Morbidly obese participants reported more absolute energy contributed by beverages (~300 kcal/day) than either obese or overweight (249 and 278 kcal/day, respectively) participants; however, percentage of energy intake from beverages was similar for overweight (19.9%) and morbidly obese (19.3%) participants. Obese participants reported 17.1% of energy from beverages. There were no significant differences in the percentage of participants reporting any (v. no) consumption of sweetened beverages by strata of demographic characteristics. Table 2 presents mean beverage consumption for categories of beverages among consumers with results stratified by BMI (overweight, obese, morbidly obese). Among participants who reported consuming sugar sweetened beverages, those who were morbidly obese reported the highest daily energy intakes from beverages (187 kcal) compared to overweight (161 kcal) and obese participants (150 kcal). Morbidly obese participants also reported a lower percentage of their total beverage intake as water (48.8% total volume (mL)) compared to either overweight (53.4%) or obese (51.7%) participants. In multiple regression analyses total energy from beverages and percent energy from beverages were not significantly associated with BMI modeled as a continuous outcome (Table 3). Similarly, no clear patterns emerged for energy intake from beverages in multinomial regression models where BMI was considered as a categorical outcome variable [10]. Finally, in multivariate regression models, none of the a priori selected grouped beverage variables were associated with BMI as a continuous outcome (Table 3) or as a categorical outcome [10].

## 4. Discussion

In this population of low-income overweight and obese African American women mean beverage energy intake was 272 (±195) kcal/day or approximately 18% of total daily energy intake. The most commonly consumed beverages were water (88.2%), sugar sweetened beverages (78.9%) and milk without additions (68.2%). 

Daily consumption of sugar sweetened beverages was common across all demographic characteristics considered in our analyses. Approximately 79% of our participants reported consuming sugar sweetened beverages, similar to the 82% observed by Bleich [11] among US Non-Hispanic Black adults. Although consumption was more common among overweight than obese participants in our study, among consumers, mean energy intake (kcal/day) from sugar sweetened beverages was highest among the morbidly obese (187 ± 135 kcal/day). This value exceeds the American Heart Association’s recommendation for no more than 100–150 kcal/day from all added sugar [12]. For those who were morbidly obese and reported consuming sugar sweetened beverages, 38% of their total fluid intake was from sugar sweetened beverages. 

In multivariate regression analyses we did not observe any associations between intakes of energy-contributing beverages (total), sugar sweetened beverages, or other categories of beverages with current weight. However, based on our data, we cannot reject the possibility that energy-containing beverages could be a contributing factor to future weight gain. In a randomized controlled crossover design including 44 women, DellaValle et al. [13] observed that energy-containing beverages consumed with a meal added to energy intake without significantly affecting satiety. Similarly, a recent systematic review concluded that energy consumed in liquid form is more difficult to offset in subsequent meals and could promote positive energy balance [7]. Energy intakes from sugar sweetened beverages of the magnitude reported in this study (e.g., 1000–1300 kcal/week), which appear relatively modest, could lead to weight gain on the order of one or more pounds per month in the context of overall energy intakes above the requirements for weight maintenance. In addition, a growing body of research suggests that regular consumption of sugar sweetened beverages may be associated with incidence of cardiometabolic disease, independent of obesity [14].

Previous research of the relationship between sugar sweetened beverage consumption and weight among adults has been summarized in a number of recent comprehensive reviews. A systematic review of sugar-sweetened soft beverages and obesity by Gibson [15] concluded that the effect of sugar sweetened beverages on obesity among adults was likely small except in susceptible individuals or at very high intakes. Gibson [15] described results from six previous reviews of sugar sweetened beverages and obesity; two citing the evidence as strong, one probable, and three inconclusive or negligible. Notably, in prospective studies the strongest associations between sugar sweetened beverage consumption and increases in weight have been observed among participants who increased their consumption over time compared to those who maintained either a high or a low consumption. Another recent review focusing on the evidence of common beliefs in obesity research stated that although increased consumption of energy-contributing beverages tends to be coupled with increased total energy intake, associations with BMI have rarely been observed [16]. In contrast, Hu [17] concluded that when all the evidence is considered, the relationship between consumption of sugar sweetened beverages and weight is modest but compelling and important from a public health perspective.

Beverage intakes are of interest beyond the contribution to energy intake. Milk is a source of key nutrients, including calcium and vitamin D, and in some emerging research has been linked with cardiovascular health benefits [18,19]. In contrast to our results where more than 2/3 of participant reported consuming milk, in nationally representative data for Non-Hispanic Black adults for 1999–2004, Bleich and colleagues [11] reported that only 31% of those ages 20–44 consumed milk on any given day (data were not stratified by gender). Vitamin C, a water-soluble antioxidant, is an essential nutrient with well-established roles in a number of biological processes including immune function, collagen synthesis, and protein metabolism. There is at least some evidence that citrus juices may have potential benefits for chronic disease prevention [17,18]. 100% fruit and vegetable juices, important contributors to vitamin C and phytochemical intake, were consumed by 40% of respondents in our study. In the study by Bleich et al. [11] 24% of Non-Hispanic Black adults 20–44 years old reported consuming 100% fruit and vegetable juices. 

This research had several strengths. We report results for consumption of total beverage and categories of beverages among a unique population of low-income overweight and obese African American women in South Georgia. Demographic data and two days of beverage intake data were collected and processed by trained staff using standardized methods. Despite these strengths, there are several limitations with our study. This population was recruited as part of an intervention study focusing on weight management; therefore, all of the participants were overweight or obese, and the results may not be generalizable to similar adults of healthy weight. This analysis is cross-sectional, and includes self-reported data for both weight and dietary intake data, and thus could be subject to confounding or bias. Although these data were collected at baseline and prior to any intervention, if obese and morbidly obese persons, had already decreased their consumption of energy-containing beverages or replaced consumption of caloric beverages with non-caloric alternatives to facilitate weight loss, then we would underestimate the relationship between beverage energy intake and weight. We are unable to completely explore this possibility with our data. Compared to overweight women (20.6%) more morbidly obese women (30.1%) reported any consumption of artificially sweetened beverage; yet, the percent of total beverage volume contributed by artificially sweetened beverages among the morbidly obese women (27.3%) was not strikingly different than overweight women (25.2%). Under-reporting of energy intake by overweight and obese persons has been consistently reported [20]. If under-reporting of energy-containing beverages was more substantial among women with higher BMIs, as a result, we could underestimate the relationship between energy-containing beverages and BMI. We cannot completely rule out this possibility; however, mean sugar sweetened beverage volume was greatest among morbidly obese participants in this population.

## 5. Conclusions

To conclude, in this study of overweight and obese African American women, we did not observe a significant association between intakes of overall energy-containing beverages or categories of beverages with current weight status. Due to the cross-sectional nature of this data we cannot estimate the prospective relationship between beverage intake and obesity incidence or weight gain. For example, we cannot rule out the possibility that energy from beverages contributed to positive energy balance and weight gain over time. In a future analysis we may further explore the potential contribution of intervention-related changes in sugar sweetened beverage consumption on weight loss. An important consideration is that our tailored intervention model allowed intervention participants to select healthy actions to focus on and not all intervention participants (*n* = 89) selected decreasing sugar sweetened beverage intake as a behavior they wished to modify. Nonetheless it is widely accepted that negative energy balance leads to weight loss. Thus, reducing consumption of beverages that contribute energy but not important nutrients (e.g., sugar sweetened beverages) could be an effective strategy for promoting future weight loss in this population.

## Figures and Tables

**Table 1:** Total Beverage Consumption (Mean and Standard Deviation -SD) and Percent Reporting Any Consumption (%) of Selected Subgroups of Beverages, by Participant Characteristics.

Characteristic	*n* (%)	Total Intake (serv.)	Total Volume (mL)	Total Energy from Beverages (kcal)	% Energy From Beverages (%)	100% Fruit/Vegetable Beverages (% Any)	Milk Beverages (No Additions) (% Any)	Non-Dairy Milk Beverages (% Any)	Sweetened, Flavored Milk Beverages (% Any)	Sweetened Beverages (% Any)	Artificially Sweetened Beverages (% Any)	Unsweetened Beverages (% Any)	Alcohol (% Any)	Water (% Any)
Sample	280 (100.0)	6.0 (2.8)	1355.2 (657.2)	272.9 (192.2)	18.3 (10.6)	112 (40.0)	191 (68.2)	7 (2.5)	10 (3.6)	221 (78.9)	82 (29.3)	131 (46.8)	16 (5.7)	247 (88.2)
Age (years) (missing = 1)														
35–50	131 (47.0)	6.2 (3.0)	1404.5 (704.3)	304.2 (203.1)	19.6 (11.4)	55 (42.0)	83 (63.4)	5 (3.8)	5 (3.8)	106 (80.9)	33 (25.2)	50 (38.2)	13 (9.9)	116 (88.5)
50+	148 (53.1)	5.8 (2.6)	1311.5 (614.0)	244.2 (178.2)	17.2 (9.7)	56 (37.8)	107 (72.3)	2 (1.4)	5 (3.4)	114 (77.0)	49 (33.1)	80 (54.1)	3 (2.0)	130 (87.8)
Education (missing = 1)														
<HS/GED	59 (21.2)	4.8 (2.5)	1088.4 (578.0)	207.9 (152.4)	15.2 (9.0)	19 (32.2)	45 (76.3)	0 (0.0)	1 (1.7)	42 (71.2)	19 (32.2)	32 (54.2)	2 (3.4)	53 (89.8)
≥HS	220 (78.9)	6.3 (2.8)	1427.0 (661.4)	290.2 (198.7)	19.2 (10.8)	92 (41.8)	145 (65.9)	7 (3.2)	9 (4.1)	178 (80.9)	63 (28.6)	98 (44.5)	14 (6.4)	193 (87.7)
Residence														
Urban	153 (54.6)	6.1 (2.8)	1389.0 (663.1)	265.9 (181.2)	17.5 (9.6)	60 (39.2)	98 (64.1)	3 (2.0)	4 (2.6)	118 (77.1)	45 (29.4)	81 (52.9)	11 (7.2)	133 (86.9)
Rural	127 (45.4)	5.8 (2.8)	1314.5 (650.3)	281.3 (205.1)	19.3 (11.6)	52 (40.9)	93 (73.2)	4 (3.1)	6 (4.7)	103 (81.1)	37 (29.1)	50 (39.4)	5 (3.9)	114 (89.8)
Living (missing = 1)														
No children	130 (46.6)	6.0 (2.8)	1358.3 (666.0)	250.8 (186.9)	17.2 (10.1)	50 (38.5)	90 (69.2)	1 (0.7)	5 (3.8)	100 (76.9)	41 (31.5)	71 (54.6)	8 (6.2)	114 (87.7)
Children	149 (53.4)	5.9 (2.8)	1343.4 (644.5)	290.2 (194.8)	19.3 (11.0)	62 (41.6)	101 (67.8)	6 (4.0)	5 (3.4)	120 (80.5)	41 (27.5)	60 (40.3)	8 (5.4)	132 (88.6)
BMI														
Overweight	34 (12.1)	5.3 (2.1)	1211.3 (506.9)	278.0 (176.9)	19.9 (11.4)	14 (41.2)	21 (61.8)	0 (0.0)	0 (0.0)	30 (88.2)	7 (20.6)	17 (50.0)	0 (0.0)	28 (82.4)
30–<40	133 (47.5)	6.0 (3.0)	1349.3 (685.8)	249.3 (192.5)	17.1 (10.1)	56 (42.1)	84 (63.2)	5 (3.8)	6 (4.5)	103 (77.4)	41 (30.8)	70 (52.6)	8 (6.0)	114 (85.7)
≥40	113 (40.4)	6.2 (2.8)	1405.5 (661.2)	299.1 (194.3)	19.3 (10.9)	42 (37.2)	86 (76.1)	2 (1.8)	4 (3.5)	88 (77.9)	34 (30.1)	44 (38.9)	8 (7.1)	105 (92.9)
Income (missing = 6)														
$10,000 or under	95 (34.7)	5.0 (2.6)	1146.6 (598.9)	247.4 (177.7)	18.7 (12.1)	34 (35.8)	63 (66.3)	1 (1.1)	2 (2.1)	73 (76.8)	30 (31.6)	42 (44.2)	3 (3.2)	80 (84.2)
$10,001–$25,000	106 (38.7)	6.4 (3.0)	1452.1 (685.5)	272.2 (182.9)	18.2 (9.7)	45 (42.5)	74 (69.8)	4 (3.8)	4 (3.8)	83 (78.3)	28 (26.4)	49 (46.2)	7 (6.6)	95 (89.6)
$25,000 or more	73 (26.6)	6.5 (2.5)	1486.6 (606.4)	303.3 (219.3)	17.9 (10.0)	30 (41.1)	49 (67.1)	2 (2.7)	4 (5.5)	60 (82.2)	22 (30.1)	39 (51.3)	6 (8.2)	66 (90.4)

Note: Standard deviation (SD) or percentage (%) in parentheses. Percentages may not add up to 100 due to rounding.

**Table 2:** Mean (SD) Beverage Volume (mL), Percent Total Beverage Volume (%) and Mean (SD) Energy Contribution (kcal) from Selected Subgroups of Beverages by BMI.

Beverage	Overweight				BMI 30–<40				BMI ≥ 40			
	% Any Use	Mean (SD) (mL) *	% Total mL	Mean (SD) Energy (kcal) *	% Any Use	Mean (SD) (mL) *	% Total mL	Mean (SD) Energy (kcal) *	% Any Use	Mean (SD) (mL) *	% Total mL	Mean (SD) Energy (kcal) *
Water	82.4%	719.1 (513.6)	53.4 (24.3)	0.0	85.7%	794.9 (623.4)	51.7 (23.0)	0.0	92.9%	755.7 (621.6)	48.8 (25.1)	0.0
100% Fruit/veg	41.2%	101.6 (88.7)	10.8 (12.1)	89.6 (52.1)	42.1%	133.1 (137.4)	11.8 (12.5)	101.6 (81.4)	37.2%	132.3 (94.0)	11.2 (9.3)	94.4 (96.6)
Milk	61.8%	64.7 (98.4)	7.7 (10.9)	155.4 (166.4)	63.2%	75.6 (118.3)	6.3 (7.1)	118.9 (106.0)	76.1%	66.3 (78.2)	5.7 (8.3)	134.9 (96.5)
Non-dairy milk	0.0	0.0	0.0	0.0	3.8%	150.2 (109.2)	11.6 (11.5)	94.2 (68.5)	1.8%	354.9 (167.3)	16.7 (1.1)	209.6 (76.3)
Milk, sweetened, flavored	0.0	0.0	0.0	0.0	4.5%	147.9 (83.1)	7.3 (3.7)	110.8 (87.9)	3.5%	170.0 (69.9)	13.8 (5.8)	123.6 (28.5)
Sugar sweetened other than milk	88.2%	418.8 (361.6)	36.1 (24.9)	161.4 (137.5)	77.4%	370.4 (294.3)	31.7 (23.8)	150.1 (123.3)	77.9%	468.9 (341.9)	37.9 (24.5)	187.1 (135.5)
Artificially sweetened	20.6%	268.9 (143.8)	25.2 (13.5)	2.9 (1.8)	30.8%	384.6 (246.5)	30.8 (19.0)	5.1 (6.4)	30.1%	408.0 (377.4)	27.3 (21.9)	5.0 (4.6)
Unsweetened not including water	50.0%	224.6 (103.7)	19.7 (9.9)	4.3 (4.2)	52.6%	268.8 (186.4)	22.0 (16.6)	3.2 (2.8)	38.9%	256.3 (176.5)	18.8 (13.5)	9.6 (38.3)
Alcohol	0.0	0.0	0.0	0.0	6.0%	99.9 (65.8)	8.4 (5.8)	193.0 (146.9)	7.1%	78.2 (74.3)	7.1 (7.8)	110.3 (90.6)

This is among users so zero’s will not be averaged in. * Standard deviation (SD) in parentheses.

**Table 3:** Regression Analyses of the Relationship between BMI (continuous outcome) with Total and Percent Energy from Total Beverages and Classes of Beverages (continuous exposures) Adjusting for Demographic Characteristics *.

Variable	Estimate (SE)	*p*-Value	Model *r*^2^	Model Pr > *F*
Total Beverage Energy (kcal)	0.0041 (0.003)	0.15	5.10%	0.05
% Energy Total Beverages (% kcal)	0.0005 (0.0005)	0.30	4.74%	0.07
Energy: 100% Fruit/Vegetable (kcal)	−0.0057 (0.008)	0.47	5.59%	0.13
Unsweetened Milks **	0.0037 (0.005)	0.45		
Sweetened Beverages ***	0.0053 (0.004)	0.19		
Alcohol	0.0103 (0.01)	0.41		
% Energy: 100% Fruit/Vegetable (% kcal)	−0.1088 (0.12)	0.35	5.72%	0.11
Unsweetened Milks **	0.0359 (0.07)	0.62		
Sweetened Beverages ***	0.0958 (0.07)	0.15		
Alcohol	0.1468 (0.24)	0.53		

* All models adjusted for age, education (<HD/GED v. ≥HS), residence (urban vs. rural), living with children (yes vs. no), income (≤$10,000, $10,001–25,000, ≥$25,000) Models use continuous outcome (BMI in kg/m^2^, estimate with standard error (SE) and continuous exposure variables (kcal, % kcal); ** Includes dairy and non-dairy milks, unsweetened; *** Includes all sugar sweetened beverages (e.g., sodas, fruit drinks, sweetened coffee/teas, milk beverages with added sugar and flavor).
